# Ten Lessons From the Spanish Model of Organ Donation and Transplantation

**DOI:** 10.3389/ti.2023.11009

**Published:** 2023-05-25

**Authors:** Simon Streit, Charlotte Johnston-Webber, Jasmine Mah, Apostolos Prionas, George Wharton, Daniel Casanova, Elias Mossialos, Vassilios Papalois

**Affiliations:** ^1^ Department of Health Policy, London School of Economics and Political Science, London, United Kingdom; ^2^ Department of Medicine, Dalhousie University, Halifax, NS, Canada; ^3^ Department of Surgery, Imperial College, London, United Kingdom; ^4^ Department of General Surgery, Whipps Cross Hospital, Barts Health NHS Trust, London, United Kingdom; ^5^ University Hospital Valdecilla, University of Cantabria, Santander, Spain; ^6^ Institute of Global Health Innovation, Imperial College, London, United Kingdom; ^7^ Renal and Transplant Unit, Hammersmith Hospital, Imperial College Healthcare NHS Trust, London, United Kingdom

**Keywords:** organ donation, organ transplantation, transplantation policy, transplant program, Spain

## Abstract

The organ donation and transplantation program in Spain has long been considered the gold standard worldwide. An in-depth understanding of the Spanish program may promote the development and reform of transplant programs in other countries. Here, we present a narrative literature review of the Spanish organ donation and transplantation program supplemented by expert feedback and presented according to a conceptual framework of best practices in the field. Core features of the Spanish program include its three-tiered governing structure, close and collaborative relationships with the media, dedicated professional roles, a comprehensive reimbursement strategy, and intensive tailored training programs for all personnel. Several more sophisticated measures have also been implemented, including those focused on advanced donation after circulatory death (DCD) and expanded criteria for organ donation. The overall program is driven by a culture of research, innovation, and continuous commitment and complemented by successful strategies in prevention of end-stage liver and renal disease. Countries seeking ways to reform their current transplant systems might adopt core features and may ultimately aspire to include the aforementioned sophisticated measures. Countries intent on reforming their transplant system should also introduce programs that support living donation, an area of the Spanish program with potential for further improvement.

## Graphical Abstract

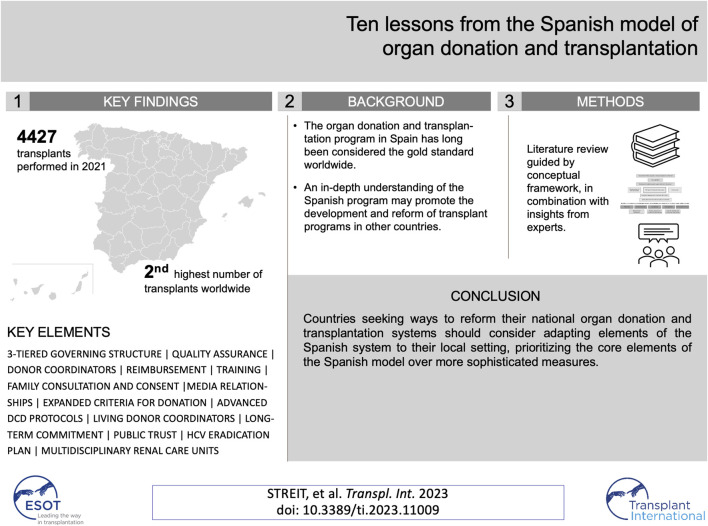

## Introduction

National transplantation rates in Europe vary substantially. Before the global COVID-19 pandemic, the number of patients receiving a transplant in the European Union ranged from 114.8 per million population (pmp) in Spain to only 7.6 pmp in Bulgaria (1). Of note, while these differences do not necessarily correlate with the availability of resources (2, 3), they do highlight the need for countries to learn from one another in order to identify ways to build better organ donation and transplantation programs.

Like other European countries, Spain has an aging demographic potentially at risk of organ failure. Additionally, high smoking rates, obesity, and alcohol consumption contribute to organ failure (Table 1). As a result, there is a substantial prevalence of people with chronic kidney disease and patients maintained on renal replacement therapy (Table 1).

**TABLE 1:** Health system financing and population health in Spain: key statistics.

Health system	References
• Mainly tax-funded national health system	(15)
• Health spending *per capita*, EUR 2488; EU average, EUR 3523	(15)
• Health spending as a percentage of the gross domestic product, 9.1%; EU average, 9.9%	(15)
• Public spending as a percentage of the total health expenditure, 70.6%; EU average, 79.7%	(15)
• Out-of-pocket payments as a percentage of the total health expenditure, 21.8%; EU average, 15.4%	(15)
• Percentage of the population reporting an unmet need for medical care, 0.2%; EU average, 1.7%	(15)
**Health status**	
• Percentage of the population over 65 years of age, 20%; EU average, 20.6%	(16)
• Life expectancy, 84 years; EU average, 80.6 years	(15)
• Percentage of the adult population that smokes daily, 19.8%; OECD average, 16.5%	(17)
• Liters of alcohol consumed *per capita* per year, 10.7L; OECD average 8.7L	(17)
• Percentage of adults that are overweight or obese (BMI >25), 50.2%; OECD average, 56.4%	(17)
• Individuals maintained on renal replacement therapy, incidence 152 pmp	(18)
• Individuals maintained on renal replacement therapy, prevalence 1,368 pmp	(18)
• Age-standardized prevalence of chronic kidney disease, 5%; global, 8.7%	(19)

EUR, Euro; EU, European Union; OECD, Organisation for Economic Co-operation and Development; BMI, body mass index.

In response, Spain has built a world-leading transplantation program with limited financial resources compared to other European countries (Table 1). Thus, a careful evaluation of the Spanish program may provide useful and important lessons for other countries. Donation rates in Spain have been the highest worldwide for many years (1, 4–6). The Spanish organ donation and transplantation organization has also taken a leading role in global efforts to improve transplant programs *via* its participation in projects that include the European Union (EU) Action Plan on Organ Donation and Transplantation and the Global Observatory on Donation and Transplantation (7, 8). Consequently, many academic publications and government reports are available that review the key policies of the Spanish transplant program (9–12). Previous analyses of the Spanish organ donation and transplantation program have highlighted features including its three-tiered system of governance, availability of ample professional teaching opportunities, comprehensive reimbursement scheme, and proactive relationships with the media (10, 12, 13). Recent reviews have also highlighted several advanced clinical protocols, including methods used to identify potential donors in locations other than intensive care units (ICUs) as well as the pursuit of expanded criteria for donation and DCD (11).

This study aims to provide an updated review of the Spanish transplant system and to assemble both existing and complementary findings within a conceptual framework that was recently developed to guide the comprehensive analysis of organ donation and transplantation programs (14). We anticipate that this effort will permit us to identify critical information that may assist other countries in efforts to develop or reform their national programs.

## Materials and Methods

This paper is based on a report that focused on the Spanish organ donation and transplantation program as part of a comprehensive document that provided information relevant to program reform in Greece (20). The manuscript presents the findings from this report that have been updated and restructured according to the best practices conceptual framework.

As a first step, we performed a narrative review of the literature focused on the Spanish transplant system. Relevant academic literature was identified by searching the PubMed database using the keywords “Spain” and “organ donation and transplantation”. Relevant grey literature was also collected from Google search, including key documents obtained from the website of the National Transplant Organization (NTO) in Spain. The literature review was complemented by an expert consultation with author Dr. Daniel Casanova, professor of transplant surgery at the University Hospital Valdecilla in Spain and former president of the transplant division of the European Union of medical specialists. In a first interview, Dr. Casanova presented key features of the Spanish system and answered open questions. In the following correspondence, he provided additional data, including family refusal rates. He also answered questions regarding clinical practices, pre-mortem cannulation, legislation, and reimbursement practices and provided additional literature for review.

**FIGURE 1:**
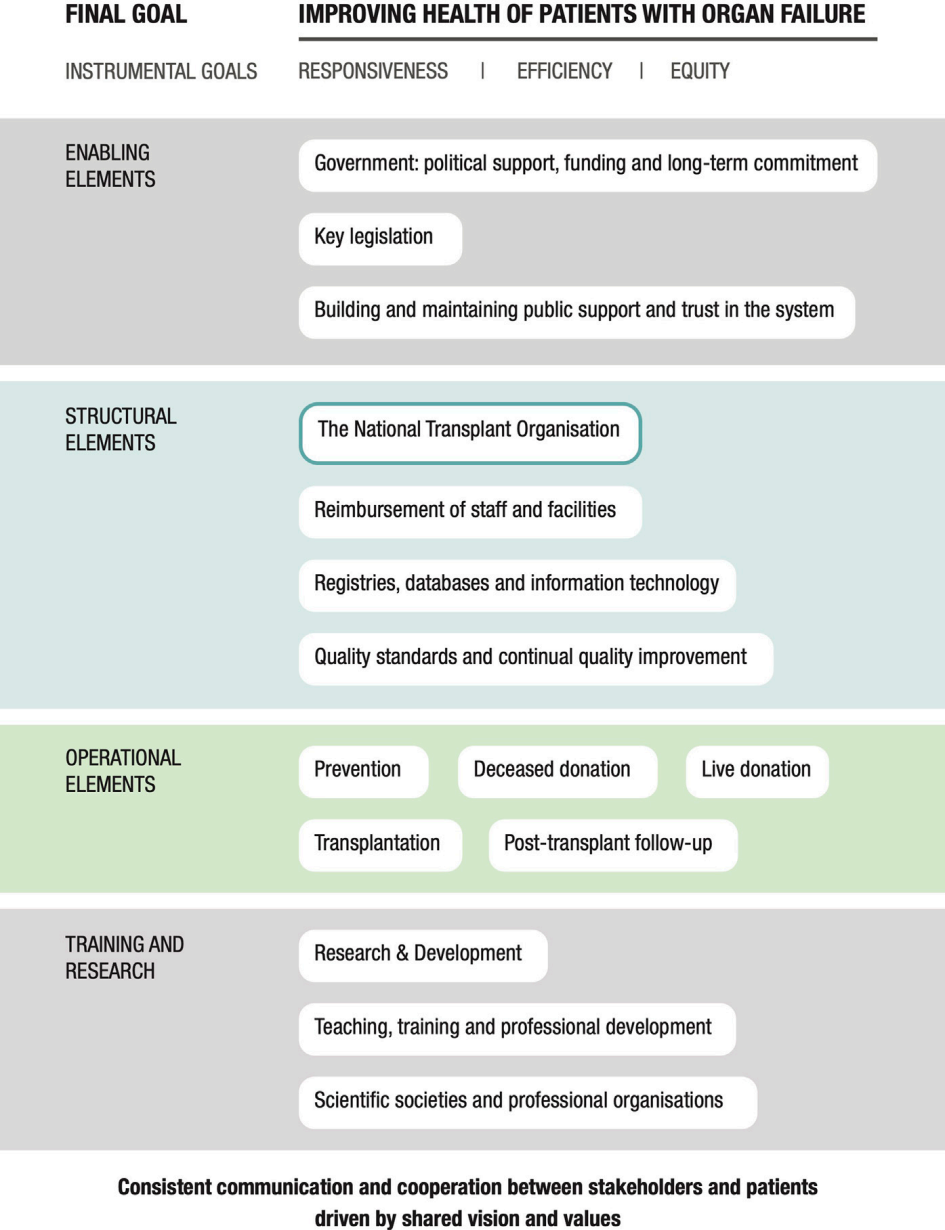
Goals and domains that build a successful transplant system (14).

The final set of findings was structured according to the organ donation and transplantation program domains described by Johnston-Webber et al. (14) (Figure 1). The analysis focused on structures, processes, and distinctive features of the system corresponding to domains of the framework. For each domain, we first present the relevant key features of the Spanish transplant system. We then suggest specific policies from the Spanish system that might be adopted by other countries seeking to develop or improve their national programs.

## Results

### Context and Trends Identified in the Spanish Transplant System

With €2,488 *per capita* spending on healthcare, the Spanish health system has fewer resources compared to the European average, both in absolute terms and relative to its economic capacity (15). In Spain, 70.6% of healthcare expenditure is based on public revenue, most of which is collected by general taxation (15). At the same time, a significant proportion of healthcare expenditure (21.8%) comes from out-of-pocket spending, mostly on pharmaceuticals (15).

Except for private providers who have been commissioned to reduce waiting lists, most healthcare services are planned and provided by the public sector (15, 21). While strategic planning and regulatory frameworks are developed at a national level, services are organized and provided by 17 regional authorities (15, 21). There is a strong emphasis on primary care with a focus on its role in gatekeeping and directing specialist care (21).

Historically, the basic organizational structure of the Spanish health system was built on the democratic Spanish constitution that was ratified in 1978 (22). The Spanish organ donation and transplantation program was initiated approximately one decade later and was built on a set of concerted reform initiatives. Major milestones in the development of the Spanish program include the implementation of dedicated institutions (1989), the introduction of DCD as part of the 40 donors pmp plan (2007), and the 50 × 22 strategic plan that further developed donation protocols promoting intensive care to facilitate organ donation, expanded donation criteria, and developed pediatric donation strategies (2018) (10–12, 23).

Largely due to these reforms, transplantation rates in Spain increased continuously and peaked at an all-time high of 5,449 transplants performed in 2019 (1). Donation and transplantation rates dropped by about 19% in 2020–4,427 transplants due to the impact of the COVID-19 pandemic (24). In the following year, the transplantation rate in Spain recovered, with 4,781 organs transplanted in 2021 (25).

Internationally, these rates are the second-highest pmp in the world and are surpassed only by transplantation rates reported in the United States of America (25). Also, Spain continues to hold the record for the highest rate of deceased donations worldwide (25).

### Key Elements and Policies Leading to Transplant Reform

The following sections present the results of the literature review that highlight ten key features of the Spanish organ donation and transplantation program which have been central to its success (Table 2). Many of these points may be adopted by other countries that are attempting to develop and/or reform their own national programs. We have also highlighted some areas of weak performance that may need to undergo further improvement.

**TABLE 2:** Ten key features of the Spanish organ donation and transplantation program that may be adopted for use by other countries. These features are displayed according to the domains of the conceptual framework proposed by Johnston-Webber et al. (14).

Framework domain	Key features	Details
Enabling Elements: Government: Political Support, Funding, and Long-term Commitment	Long-term continuous governmental commitment and support for the program	Continuous commitment to ongoing reform and development over several decades has led to sustained success
Enabling Elements: Government: Legislation	A comprehensive legal framework accompanied by acceptable clinical protocols	Spain’s well-established legal framework is complemented by clinical protocols that are acceptable to the general public. This is believed to have contributed to the high rate of deceased donations
Enabling Elements: Building and Maintaining Public Trust in the System	Policies in place that nurture a culture of trust and confidence in the organ donation and transplantation program	Extensive training of health professionals in communication skills, an excellent relationship with the media, and a focus on family consent are all factors that have helped to inspire public trust in the national program
Structural Elements: National Transplant Organization	Institutions specifically dedicated to donation and transplantation are developed and maintained on the national, regional, and hospital levels	Spain has implemented a three-tiered governing structure that encompasses the national, regional, and local levels. This may have contributed significantly to the program’s success
Structural Elements: Quality Standards and Continual Quality Improvement	Continual quality assurance has been identified as a core strategy of the Spanish program	Standardized evaluation and reporting of donation activity, as well as both internal and external audits, are performed on a rolling basis. Performance metrics from individual hospitals are compared to one another
Structural Elements: Reimbursement of Staff and Facilities	There must be no financial barriers to organ donation or participation in transplantation activities	Spain has recognized the critical link between organ donation and reimbursement. Hospital budgets include specific funding for organ donation based on the previous year’s activity
Operational Elements: Prevention	Broad public health measures and specialist policies must be developed to prevent end-stage organ failure	Spain has implemented policies that focus on limiting access to tobacco, improving food labeling, and promoting healthy eating. Multidisciplinary specialty clinics are tasked with providing care for patients with end-stage renal failure and a concerted national strategy was successfully implemented to reduce the prevalence of Hepatitis C. Spain might improve its efforts focused on strengthening secondary preventative measures in renal care
Operational Elements: Donation and Transplantation	Spain is currently applying strategies that facilitated deceased donation to improve rates of living donation	Spain has created a living donor coordinator role with clear and specific responsibilities similar to those of deceased donor coordinators
Training and Research: Teaching, Training, and Professional Development	Tailored training for professionals will provide essential skills, notably those needed for family consultations	Comprehensive, tailored teaching is an integral part of the Spanish program that most likely contributed significantly to its success. Spain offers both public and private training institutions that train all healthcare personnel involved in organ donation and transplantation
Training and Research: Research and Development	Foster a culture of innovation focused on strategy, technology, and the law	Expanding DCD has been identified as an important strategy to increase the donor pool. Advanced DCD protocols have been implemented that are supported by comprehensive legislative reform and clinical guidance

### Enabling Elements

#### Government: Political Support, Funding, and Long-Term Commitment

##### Spain Illustrates the Rewards of Long-Term Commitment to Transplantation Policy

The Spanish organ donation and transplantation program has undergone continuous development over the past four decades. While key legislation initially established the program in 1979, core features of the “Spanish model”, including the appointment of donor transplant coordinators, development of training opportunities, and the three-tiered governing structure were all established over the 30 years that followed (10–13). Initial reforms focused on deceased donation after brain death (DBD) (10–13). Further reforms focused on several advanced clinical protocols including DCD, efforts to identify possible donors from sources other than ICUs, as well as expanded donation criteria (11). Finally, efforts to increase the rate of deceased donation have been complemented by increased activity in the field of living donation (23, 24).

These developments highlight the continuous commitment of the stakeholders in the Spanish transplant system and their ongoing efforts to improve the system even after achieving world leadership. Countries seeking to reform their transplant systems must be aware of the long-term commitment that is necessary to achieve sustainable success.

#### Government: Key Legislation

##### A Basic Legal Framework Must Be Complemented by Effective Clinical Protocols

As in other European countries, Spain has built a solid legislative framework that supports its institutions and consent policy as well as the regulation of the different modes of donation in a set of laws and royal decrees (25). From a purely legal standpoint, all Spanish citizens who have not specifically stated their unwillingness may be considered for organ donation. However, in clinical practice, donations are only pursued after family consultation and approval (26). This illustrates the fact that, despite the popularity of presumed-consent legislation, its importance should not be overemphasized. Thus, although a legislative framework is an essential component of a successful system, legislation must be complemented by protocols that are acceptable to the general public and professionals at a working level.

#### Building and Maintaining Public Support and Trust in the System

##### The Spanish Model Promotes High Donation Rates and a Transplant System That Meets Donor and Family Expectations

In Spain, the transplant system builds on a generally positive attitude towards donation which is more favorable than the European average and has stayed consistent across different surveys for the past 30 years (26–31). Additionally, results from a recent study revealed a high level of trust specifically in the transplant system; the number of Spaniards who report a lack of trust in the national organ donation and transplantation program as a reason for opting out of organ donation is below the European average (27). Accordingly, the family refusal rate is considerably lower than that reported in other countries (32). These statistics reveal the success of the Spanish program not only in terms of the number of organs transplanted but also in gaining the confidence of donors, families, and the general public.

The success of the Spanish donation and transplantation program can most likely be attributed to policies that focus on trust and transparency. These include policies that support training opportunities for healthcare professionals that are focused on communication skills, family consultation, and consent (9, 28), direct communication with the media, including educational programs for journalists, and round-the-clock availability for consultation (29). Likewise, Spanish policy supports a conservative consent policy and practice that focuses on the needs of donor families (26).

Despite these efforts, some knowledge gaps among the general population regarding organ donation and population remain. For example, a recent survey indicated poor knowledge and consent for donation when individuals were provided with actual clinical scenarios of organ donation (30). Also, surveys revealed a lack of knowledge regarding current consent legislation both recently and in past years (30, 31). However, as indicated by the low rates of family refusal rates, these are issues that are successfully addressed in family consultations.

Taken together, building on a generally favorable attitude towards donation, the Spanish system has focused on preventing misconceptions and mistrust in the system by targeting the media and donor families directly rather than investing in broad awareness and education campaigns (26).

Other countries seeking to develop or reform their national programs might focus on building a similar culture of trust and transparency, as this is clearly essential for gaining the confidence of the general population and supporting high rates of organ donation.

### Structural Elements

#### The National Transplant Organization (NTO)

##### Institutions Specifically Dedicated to Organ Donation and Transplantation Are Needed at the National, Regional, and Hospital Levels

Spain has implemented a three-tiered governing system that oversees this process (9). On a national level, the NTO is responsible for analyzing national developments in organ donation and transplantation, building a general national strategy in cooperation with relevant stakeholders, and implementing relevant regulations and guidelines (9, 10, 23). The NTO also coordinates transplantation logistics and provides 24-h support for healthcare professionals with information focused on donation protocols and regulations (9, 10). Spain also maintains 17 regional offices that reflect the distribution of autonomous regions as well as the structure of the health system in general (21, 33). The regional offices support strategic reform processes and coordinate organ transport (21). Finally, “donor transplant coordination units” have been implemented in Spanish donation hospitals (9, 10). These units include nurses and physicians, often with a background in intensive care medicine, who have been trained to carry out this responsibility and are accredited for their role in coordinating donation activity at the hospital level (9, 10, 34). These individuals are tasked with training other clinical staff members, identifying possible donors, evaluating medical suitability for donation, documenting donation activity, consulting with relatives, and coordinating the overall clinical pathway of donation (9, 10).

This three-tiered governing structure is currently considered to be a major contributor to the success of the Spanish model; it has been used as a framework for several other successful European organ donation and transplantation programs, for example, those currently in place in the United Kingdom, Portugal, and Italy (2, 3, 35, 36).

#### Quality Standards and Continual Quality Improvement

##### Standardized Evaluation and Reporting of Donation Activity as a Strategy to Improve Quality

An integral part of the Spanish organ donation and transplantation program is the national quality and benchmarking system led by the NTO (9). Hospitals are externally audited and donor transplant coordinators periodically collect and report on a set of indicators of donation activity. The data are reported for each autonomous region as a means to encourage accountability (5, 9), and differences in hospital performance are compared in an attempt to identify the potential for improvement at a local level as well as areas in need of strategic national reform (11). Continuous quality assurance is understood to be an essential component of the Spanish organ donation and transplantation program (11). Countries aiming to reform their programs should consider periodic quality evaluations, reporting, and feedback as vital strategies that might be developed to ensure continuous improvement.

#### Reimbursement of Staff and Facilities

##### Policymakers Should Review Reimbursements for Donation Activity in Order to Identify Any Financial Barriers to Participation

Similar to other medical procedures, donation and transplantation activities must be appropriately reimbursed and there should be no financial barriers to implementing and participating in these activities (14). Spanish officials have highlighted the critical link between organ donation activity and reimbursement. Hospital budgets in Spain are provided with funds to cover the costs of donation activity based on previous donation rates (13). Accordingly, countries seeking to reform their transplant systems should revisit their national reimbursement practices and identify potential financial barriers to donation.

### Operational Elements

#### Prevention

##### Broad Public Health Policies and Specialty Care Models Should Be Used to Address End-Stage Renal Failure

As part of a wider public health initiative designed to reduce cardiovascular risk factors, Spain has limited access to tobacco products and improved both labeling and promotion of healthy foods (15). As cardiovascular risk factors are highly relevant to organ failure, most notably, renal disease (37, 38), these initiatives are promising from the perspective of the national transplantation program. Nonetheless, secondary programs focused on the prevention of organ failure need further improvement. In particular, current problems include comparatively late-stage referrals from primary to specialist care as well as suboptimal management of diabetes and arterial hypertension (39, 40). This is illustrated by comparably low rates of screening for high blood pressure (41). We recognize that these conditions may have developed given the increased pressure placed on the primary care system due to the overall increase in chronic conditions as well as budgetary constraints (15).

By contrast, the health system in Spain takes an innovative approach to tertiary prevention of end-stage renal disease. Specialty care for end-stage renal disease is organized in dedicated facilities known as “UERCA units.” These units promote a multidisciplinary and quality-driven approach to this condition, including standardized protocols for transplant evaluation (42, 43).

Similarly, Spain has taken a multi-faceted approach to the prevention of end-stage liver disease. Following market access of novel antiviral drugs, the Ministry of Health developed a strategic plan that covered monitoring, prevention, and treatment of Hepatitis C Virus (HCV) infection (44). Specifically, the plan included a treatment registry and seroprevalence study, training programs for health professionals, promotion of harm reduction policies, clinical recommendations for HCV screening in primary care, patient guidance, clinical criteria and prioritization for antiviral treatment, and funding agreements (44). Efforts were coordinated by a dedicated committee of relevant stakeholders, combined with a detailed timeframe and performance indicators designed to monitor the success of this strategy (44). Strategies that include implementation and prioritization of treatment for patients with end-stage liver disease (including those on a transplant waiting list) have clearly met with success. Following the introduction of the strategic plan, both HCV-related hospitalizations and the number of patients on the liver transplant waiting list have significantly decreased (45–47).

Spain’s implementation of broad public health policies, a specialty care model for end-stage renal failure, and a dedicated strategy focused on eradicating HCV infection are important elements of the national organ donation and transplantation program that should be adopted by other countries. While the Spanish healthcare system, in principle, maintains a strong focus on primary care (15), further progress is needed to address secondary prevention of renal failure. Nonetheless, a strong, interconnected primary care system is crucial to the efforts to prevent organ failure and thus reduce the burden on the organ transplantation program.

#### Donation and Transplantation

##### Applying Successful Strategies Used to Promote Deceased Donation to Encourage Living Donation

Living kidney donation rates in Spain lie slightly above the European average (25). Spanish authorities have identified several barriers to living donation including an overall lack of professional knowledge regarding the need for living donation, poor communication with patients, and a lack of knowledge regarding modern surgical techniques (48, 49). Currently, living donation is hampered by the limited coordination between transplant centers, few to no standardized protocols, and insufficient data available to address the overall process (49, 50). Key strategies have been implemented that are designed to overcome these barriers. These strategies include providing additional training opportunities, as well as creating professional guidelines and patient information materials; defining clear responsibilities for living donation coordinators; and implementing standard protocols for patient consultation, evaluation, and referral (48–50). Spain has also established a national and international cross-kidney exchange program and supported public campaigns designed to promote living donations (51, 52).

Thus, specific strategies that have worked well in efforts to promote deceased donation (i.e., assigning specific responsibility for program coordination, offering tailored training opportunities, and placing an emphasis on patient communication) have now been applied to the process of living donation. Strategies that prove to be successful may be considered and adopted by other countries seeking to reform their living donation practices.

### Training and Research

#### Teaching, Training, and Professional Development

##### Efforts to Train Professionals Are Essential, Especially with Respect to Family Consultation and Communication Skills

Training healthcare professionals involved in organ donation and transplantation is particularly important in Spain (9, 53). Alongside private training institutions, specific public funding is dedicated to the training and accreditation of all professionals that participate in organ donation and transplantation (9, 34, 54). Training modules cover both specific steps along the clinical pathway of organ donation, including donor maintenance, as well as more general topics such as interacting with the media (9). Special emphasis has been placed on training donor transplant coordinators on how to communicate effectively with relatives. These efforts are believed to have contributed significantly to low rates of family refusal in Spain (9, 26). In summary, comprehensive and sustained teaching and training of professionals is another key component of the Spanish organ donation and transplantation program. Other countries should seek to implement national training efforts (and accreditation) or support training resources already available.

#### Research and Development

##### Fostering a Culture of Innovation Focused on Strategy, Technology, and the Law

The Spanish organ donation and transplantation program maintains an innovative spirit that can be illustrated by recent advances in the development of protocols designed to encourage DCD. Strategically, DCD donation has been identified as a means to expand the donor pool in Spain (11). Simultaneously, in pursuit of overcoming the technical limitations of DCD and improving graft survival, Spain has pioneered research in the field of normothermic regional perfusion. Transplant professionals apply pre-mortem heparinization to facilitate machine perfusion, use pre-mortem cannulation to monitor brain blood flow, and provide mobile ECMO units to local hospitals (55, 56). Cohorts of patients transplanted with normothermic regional perfusion are currently undergoing follow-up with promising preliminary results (55, 56). These technological advances have been supported by reforms that have legalized DCD and specified post-mortem intervals and clinical protocols to be used in these circumstances (55). Building on the pre-existing infrastructure in donation and transplantation, these advances have provided support for complex clinical protocols in which patients are transferred from emergency treatment into organ donation pathways within relatively short periods of time (55). Countries seeking to reform their organ donation and transplantation programs may wish to adopt some of these protocols. However, everyone needs to be aware of the technical, legal, and procedural preconditions that need to be in place in order to pursue these sophisticated donation pathways in a fully ethical manner.

## Discussion

The use of a systematic framework approach demonstrates clearly that the Spanish organ donation and transplantation program offers many examples of best practices across multiple domains. The findings in this review are consistent with previous evaluations of the Spanish system that have emphasized its leading role in organ donation and transplantation policy (9–11, 13). These findings also reflect those from studies that have highlighted successful adaptations of the Spanish program in both high- and low-resource settings (2, 3, 47).

This review also adds dimensions of the Spanish system that have not been integrated into previous reviews of the Spanish system. This study is the first to emphasize disease prevention of organ failure as a vital part of the organ donation and transplantation program in Spain. Despite successful prevention strategies for end-stage liver and renal disease and the large number of organs transplanted, there remains substantial demand. For example, the number of kidney transplants performed in 2019 (74 pmp) accounted for only 5.5% of the patients who began dialysis care during the same year (8, 18). This point illustrates the great importance of implementing demand-side measures designed to reduce the rate of end-organ failure and provides a new perspective on the Spanish transplant system.

Another novel aspect of this review is that it highlighted recent efforts to implement living donation policies. Although the Spanish program’s focus on continuous reform is clearly reflected in its living donation policies, current performance falls behind countries such as Turkey, which has achieved exceptional rates for living donation through a combination of financial commitments, education initiatives, and integration of the private sector (48). Overall, this study synthesizes existing lessons learned from the experiences of the Spanish transplant system and also highlights elements that have not been the focus of previous analyses.

Although comprehensive in its approach, the review has several limitations. First, important dimensions of transplant systems, including information technology, infrastructure, and the role of professional societies are not specifically covered in this review. ICU capacity has been discussed in the literature as a factor to be considered when adapting the Spanish model for use by other countries (13, 57). Correspondingly, the importance of ICU capacity in the Spanish context was illustrated during the COVID-19 pandemic when donation rates fell sharply due to ICU occupancy by infected patients and shortages of healthcare personnel (58, 59). However, internationally, success in organ donation and transplantation does not appear to increase in linear proportion with ICU capacity. For example, although Germany has the highest ICU capacity of all OECD countries and has significantly more ICU beds than Spain, it has not achieved similar success in organ donation and transplantation (1, 60). By contrast, Croatia has shown great success in organ donation despite its low ICU capacity compared to other European countries (1, 61, 62). Taken together, it seems that while sufficient baseline ICU capacity in Spain has contributed to its success, this factor alone does not suffice. Future studies might consider the importance of ICU capacity as well as information technology and the role of professional societies in Spain in greater detail.

In conclusion, countries seeking to reform their organ donation and transplantation policies can learn from one another using Spain as a leader and a role model. Dedicated institutions, quality assurance processes, detailed reimbursement schemes, and comprehensive training programs are all crucial features that other countries might adopt while adapting them to their specific needs. The highest priority should be given to these areas, as these have served as critical foundations of the Spanish system and have worked well in other settings, including those with fewer resources (2, 35). Countries may be capable of achieving even higher rates of organ transplantation by fully exploiting the possibility of living donation and seeking additional input designed to direct policy reform in this area.

Once these measures have been implemented, public trust has been gained, and the supporting infrastructure has been deemed to be sufficient, the more sophisticated features of the Spanish program, including innovative DCD protocols, expanded criteria for donations, and admittance to ICU for donation purposes can also be adopted.

Of note, consent policy and broad public awareness campaigns have played a smaller role in the Spanish system. These areas of policy reform might be deprioritized in countries aiming to reform their transplant systems. Finally, the Spanish example illustrates that efforts to strengthen primary care and improve primary, secondary, and tertiary prevention of end-organ disease must be perceived as integral components of any organ donation and transplantation program. Investment in these areas might ease the high demand for organ transplantation in Spain as well as in other countries.

## Data Availability

The original contributions presented in the study are included in the article/supplementary material, further inquiries can be directed to the corresponding author.
